# Gender Difference and Characteristics Attributed to Coronary Artery Disease in Gaza-Palestine

**DOI:** 10.5539/gjhs.v5n5p51

**Published:** 2013-05-26

**Authors:** Amal Jamee, Yehia Abed, Marwan O. Jalambo

**Affiliations:** 1 Cardiology Department, Al-Shifa Hospital, Ministry of Health, Palestine; 2 Faculty of Public Health, Al Quds University, Palestine; 3 Foods and chemicals Products Laboratories, Ministry of National Economy, Palestine

**Keywords:** risk factors, coronary artery disease, gender difference, Gaza

## Abstract

Traditionally coronary artery disease (CAD) has been considered as disease affecting men, and for long time women were not included in researches programme. In both sexes, coronary heart disease risk increases with age. Extensive clinical and statistical studies have identified serial factors that increase the risk of coronary heart disease, some of them can be modified, and some cannot. This study was performed to analyze the extent to which cardiovascular risk factors can explain the gender difference in coronary heart disease.

**Methods:**

The study design is a cross sectional study based on 155 cardiac patients admitted to cardiology department in Al-shifa hospital Gaza. The following cardiac risk factors were determined from the patient's records, smoking, diabetes, high blood pressure, Dyslipedemia and presence of family history of coronary artery disease. Catheterization results review were done. Statistical Package for Social Science version 17 was used for data entry and analysis. Frequency and cross tabulation were done to explore the relationship between the study variables. Chi-square test was used for testing statistical and P-value less than 0.05 were considered as significant.

**Results:**

Most of risk factors were more favorable in females and increase with age. Myocardial infarction in male compared with female was 2 times higher, and chronic angina pain is common in female than male respectively 71.4% and 46.7%. Around 77% of female have two vessels disease and more. No great differences in number of diseased vessels among patients with myocardial infarction or chronic stable angina. Patients with low EF<50% have higher chance of affected vessels (82.9%).

**Conclusion:**

CAD stay the major problem in male and female, certain patient's characteristics and clinical conditions may place female at higher risk of coronary artery disease development or progression. This article addresses emerging knowledge regarding gender differences in CAD risk factors and responsiveness to risk reduction interventions.

## 1. Introduction

Coronary artery disease (CAD) is a complex disease caused by reduced or absent blood flow in one or more of the coronary arteries that supply oxygen rich blood to the heart muscle and carry away oxygen-depleted blood. The coronary arteries consist of two main arteries: the right and left coronary arteries (Khaki et al., 2010). Coronary angiography provides a precise anatomic delineation of the presence or absence of coronary artery disease (Antoni et al., 1995), during life allows risk factors to be correlated with the precise diagnosis (Holmes et al., 1981). Cardiovascular disease is responsible for more deaths in men and women than any other cause of death in the United States and in most developed countries (Lawton, 2011).

Multiple risk factors such as hypercholesterolemia, hypertension, and diabetes mellitus are known in the origin of CAD (Acarturk et al., 2004). Acarturk et al. (2004) said that high TG concentrations were associated with a 30% and 75% rise in cardiovascular risk in men and women, respectively. Smoking is a recognized risk factor for coronary artery disease (CAD). A study entitled “Smoking in Saudi Arabia and its relation to coronary artery disease” concluded that there is a clear association between cigarettes smoking and CAD particularly among males (Al-Nozha et al., 2009). A large population has established a firm relationship between gender difference, clinical risk factors, and the development of clinical manifestation of coronary artery disease, such as cigarette smoking, sex, age, Hypertension, diabetes, and dyslipedemia (Roeters et al., 2000; Vera et al., 2001; Younes et al., 2009). Differences between identified groups at higher risk for later occurrence of angina, myocardial infarction, cardiac death are studied (Murabito et al., 1993).

Significant sex differences exist between men and women with regard to coronary artery disease. The epidemiological studies show that incidence of coronary artery disease is greater in male than female (Greenland et al., 1991; Vera et al., 2001; Jeanine et al., 2002). Miller et al. (2001) reported that the gender differences in patients with CAD have been reported to be lower or no different in women than in men. A study conducted in Turkey in 2004 found that TG was an independent risk factor for CAD only in women. The study also found that there was a positive correlation between TG concentrations and number of the diseased vessels only in women. The same study concluded that high TG concentrations increase CAD risk more in women than in men in Southern Turkey (Acarturk et al., 2004). The search for physiological reasons for differences in cardiovascular disease between women and men has included intense laboratory investigation—particularly focused on estrogen. Estrogen is thought to be beneficial because of effects on atherosclerotic plaque progression, vasodilatation, blood pressure, and its antioxidative and anti-inflammatory properties (Lawton, 2011). This study was performed to analyze the extent to which cardiovascular risk factors can explain the sex difference in coronary heart disease.

### 1.1 Study objectives

1) To identify major risk factors for coronary artery disease classified by gender.
2) To estimate the relation between numbers of significant disease vessel diagnosed on coronary angiography and multiple risk factors.
3) To analyze whether any gender related difference in patients with coronary artery disease can explain the difference of the disease incidence among male and females.

## 2. Methodology

The study design is cross sectional study based on the medical files of 155 cardiac patients admitted to cardiology department in Al -Shifa hospital - Gaza, during the year 2010. Abstract sheet is formed to gather the relevant variables from the patient records. These variables include socio demographic variables, clinical findings and cardiac risk factors. The following cardiac risk factors include smoking, diabetes, high blood pressure, dyslipedemia and presence of family history for coronary artery disease. Catheterization results review were done and recorded. Statistical package for Social Science version 17 was used for data entry and analysis. Data cleaning was done and frequency distribution was completed. Recoding for age to 2 groups and crosstab were done to explore the relationship between the study variables. All the risk factors are classified by sex to examine for the differences between males and females. Chi-square test was used for statistical testing and p value less than 0.05 were considered significant. To control for age as confounder stratification by age groups was performed.

## 3. Results

### 3.1 Distribution of the Study Population

During the study period, 155 cardiac patients with age more than 40 years diagnosed as coronary artery disease are followed and their distribution is shown in Table 1. Around 77.4% are males and 22.6% of the patients are females. More than half of the cases 62.6% are aged 40-60 years old, and those who are older than 60 years were 37.4%. By increasing, age the proportion of females increase from 30% to 45.7% of the study population.

**Table 1:** Distribution by gender and age

Gender	Age (40-60) years	Age >60 years	Total (%)
Male	78	42	120 (77.4)
Female	19	16	35 (22.6)
Total (%)	97 (62.6)	58 (37.4)	155 (100)

#### 3.1.1 Risk Factors for Heart Disease

From the patients records a group of risk factors for cardiovascular were abstracted and reported in Table 2. These factors include smoking, hypertension, diabetes mellitus, dyslipedemia and family history of coronary artery disease (FHCAD). Smoking is limited for males where no single female is considered a smoker and the percentage of smoking was 60.8% of all males with significant P-value < 0.001. Hypertension and diabetes showed high prevalence in female than male with significant level for hypertension (*P* = 0.041). Dyslipedemia and family history of coronary artery disease showed no great difference in both gender, to control for age as confounder we conducted stratification between gender and different risk factors by age group. For smoking younger males smoke more than elders and females don’t smoke. In both age groups, result is statically significant. Older patients in both gender expressed higher prevalence of hypertension, diabetes, and Dyslipedemia. In both age group females expressed higher prevalence of hypertension and diabetes more than male, but did not reach statistical significant level, for Dyslipedemia the prevalence is higher among male in both groups.

#### 3.1.2 Cardiac Status of Patients

In this study, cardiac status was measured by clinical diagnosis of patients and by the results of coronary angiography as shown in Table 2. Chronic angina pain is more common in female than male respectively 74.3% and 46.7%. Myocardial infarction was two times higher in male than female in admitted patients 53.3% and 25.7%; this difference is statistically significant (*P*-value = 0.004). Myocardial infarction is higher in males in both age groups, which is statistically significant with those who were more than 60 years. Chronic angina is more prominent in older age especially among female, which is statically significant *P*-value 0.03.

**Table 2:** Risk factors and clinical status of patients

Risk factors	Age group(years)	Male		Female		Total		*P*-value
		NO.	%	NO	%	NO	%	
Smoking	40-60	50	64.1	0	0	50	51.5	0.00
	More than 60	23	54.8	0	0	23	39.7	0.00
	Total	73	60.8	0	0.0	73	47.1	0.00
Hypertension	40-60	38	48.7	12	63.2	50	51.5	0.26
	More than 60	28	66.7	14	87.5	42	72.4	0.11
	Total	66	55.0	26	74.3	92	59.4	0.04
Diabetes	40-60	37	47.4	11	57.9	48	49.5	0.41
	More than 60	21	50	12	75	33	56.9	0.09
	Total	58	48.3	23	65.7	81	52.3	0.07
Dyslipedemia	40-60	25	32.1	6	31.6	31	32	0.99
	More than 60	21	50	6	37.5	27	46.6	0.39
	Total	46	38.9	12	34.3	58	37.4	0.66
FHCAD	40-60	37	47.4	6	31.6	43	44.3	0.21
	More than 60	18	42.9	7	43.8	25	43.1	0.95
	Total	55	45.8	13	37.1	68	43.9	0.36
Myocardial infarction	40-60	43	55.1	6	31.6	49	50.5	0.07
	More than 60	21	50	3	18.8	24	41.4	0.03
	Total	64	53.3	9	25.7	73	47.1	0.00
Chronic angina	40-60	35	44.9	13	68.4	48	49.5	0.06
	More than 60	21	50	13	81.3	34	58.6	0.03
	Total	56	46.7	26	74.3	82	52.9	0.004

### 3.2 Relationship between Vessels Diseased, Risk Factors and Cardiac Status

In this part of the results, the researchers explored the relationship between the different cardiovascular risk factors and the affected vessels. Table 3 showed that more than two third (70.3%) of patients in the study population have two vessels disease and more with high percentage among female (77.1%). There is no difference about one vessel disease between males and females, but left main stem disease is three times higher in males (15.0%). It is remarkable that more than two thirds of patients who have diabetes mellitus (DM), hypertension (HT), smoker, dyslipedemia and FHCAD have more than two vessels disease; the most important risk factors in-group with one vessel was smoker and in left main stem disease was dyslipedemia and diabetes.

**Table 3:** Relation between risk factors and affected vessels

Categories	Male		Female		Smoker		DM		HT		FHCAD		Dyslip.	
	No	%	No	%	No	%	No	%	No	%	No	%	No	%
1 vessel	20	16.7	6	17.1	16	21.9	9	11.1	14	15.2	10	14.7	4	6.9
≥2 vessels	82	68.3	27	77.1	50	68.5	61	75.3	69	75	50	72.5	45	77.6
Left main stem	18	15.0	2	5.7	7	9.7	11	13.6	9	9.8	8	11.8	9	15.5

The researchers demonstrated the relation between the affected vessel and the clinical diagnosis as shown in Table 4 shows that the occurrence of myocardial infarction is associated with the number of the vessels affected, in patients with myocardial infarction presentation 72.6% have two vessels and more while this percentage was 68.3% for stable angina patients. The difference is not statistically significant (*P*-value = 0.69). Patients with low EF < 50 % have higher chance of affected vessels. The differences is statistically significant *P*-value ≤ 0.001

**Table 4:** Relation between clinical diagnoses, Ejection fraction (EF) and diseased vessels

Diagnostic	1 vessel		≥2 vessels		LM stem		Total	
	NO	%	NO	%	NO	%	NO	%
Myocardial infraction	11	15.1	53	72.6	9	12.3	73	100
Stable angina	15	18.3	56	68.3	11	13.4	82	100
EF>50%	24	20.0	80	66.7	16	13.3	120	100
EF<50%	2	5.7	29	82.9	24	11.4	35	100

## 4. Discussion

Despite major advances, in the diagnosis and treatment of heart disease, coronary artery disease remains the leading cause of morbidity and mortality in both men and women in worldwide (Steingart et al., 1991; Younes et al., 2001; Jeanine et al., 2002). The literature showed that there are differences between the gender in the prevalence and impact of coronary heart disease risk factors (Holmes et al., 1981; Greenland et al., 1991; Castanho et al., 2001; Khaki, 2010). In this study population, there were significant differences between male and female concerning cardiovascular risk factors, so cigarette smoking is very prevalent in young group and limited for males. We observed that hypertension and diabetes are present at high level in women than men, and the percentage of hypertension, and diabetes increase in both gender with age. In this study, we try to conduct for age by stratification, and our findings revealed that the differences between male and females in myocardial infarction and chronic angina remains constant, while differences in the risk factors between males and females showed higher prevalence among female and this difference did not reach a statistically significant level.

In USA for example some risk factors have high prevalence among female in the 20-74 years age group, more than 1/3 have hypertension and more than1/4 have Dyslipedemia (Castanho et al., 2001; Levit et al., 2011). In this study, the results consistent with the results report from the American heart association by Lloyd et al., in 2010, also a study conducted in Turkey in 2004 found that TG was an independent risk factor for CAD only in women. The study also found that there was a positive correlation between TG concentrations and number of the diseased vessels only in women. The same study concluded that high TG concentrations increase CAD risk, more in women than in men in Southern Turkey (Acarturk et al., 2004). In our study women are more likely than men to have chronic stable angina (74.3% for females vs. 46.7% for males) in contrast myocardial infarction was two times higher in males than females (53.5% in male vs, 25.7% in female). Also in this study there is a significant relation between the number of diseased vessels and risk factors, so 68.5% of smoker, 75.3% of diabetic 75% of hypertensive, 77.6% of dyslipedemia and 72.5% with positive family history of coronary artery disease patients have more than two vessels disease.

In a report of a clinical study in Netherlands over 16 years period 1981-1997 evaluated 1894 patients with coronary angiography documented showed no gender difference in extent of coronary lesion observed (Roeters et al., 2000). This study found that there is no great difference in the number and extent of diseased vessels in both gender and in-group with more than two vessels diseases. Knowing that the risk factors are more present in females than males, the incidence of one vessel disease was similar in males and females, but the left main stenosis was present three times higher in men (15%-5.7%). Patients with low left ventricular EF have multi-vessels coronary disease (82.9%); in contrast, patients with EF more than 50% have one vessel disease (20%). The same results were found in a study in Imam Khomeini hospital in IRAN (Younes et al., 2009). It showed no gender differences in the number of diseased vessels with higher prevalence of risk factors such as diabetes mellitus and hypertension in women.

## 5. Conclusion

The results of this study indicated that gender has an important influence on coronary artery disease, and the researchers recommended risk factors modification and primary prevention of coronary atherosclerosis in our population in Gaza. Finally, an increase in educational message focusing on cardiovascular disease promotes the overall objectives of enhancing the cardiovascular health of male and women in our country.
